# Supplementary material for: Cranial functional specialisation for strength precedes morphological evolution in Oviraptorosauria
Source: Commun Biol. 2024 Apr 10;7:436. doi: 10.1038/s42003-024-06137-1 (PMC11006937; doi:10.1038/s42003-024-06137-1)
Supplement: Supplementary file 2 — Supplementary Information [file 42003_2024_6137_MOESM2_ESM.pdf]

Supplementary Text S1: Additional FEA scenarios

Methods

A number of further scenarios using extrinsic loading were created to model a 'head-pull', 'head-shake', and a 'head-twist' motion (similar to Montefeltro *et al*., 2020) to additionally test the performance of the crania in ways that may be relevant to feeding, driven by postcranial musculature. The pull scenario comprised two anteriorly directed loads on the lingual surface of the beak tip/front teeth. The shake scenario comprised two loads directed right laterally, one on the medial edge of the right mid-palate tooth/beak rim and one on the opposite lateral edge of the left. The twist scenario comprised a dorsally directed load on the bottom of the right mid-palate tooth/beak rim, and another load ventrally directed at a node corresponding to the region of the premaxilla/maxilla directly above the same point on the opposite side. Models were constrained at the quadrate and occipital condyle for these extrinsic scenarios. Extrinsic loads for pulling, shaking, and twisting scenarios were scaled to cranial surface area but half the value of those in the comparative bending test (as these produced mean von Mises stresses in the pull, shake, and twist scenarios of similar magnitude to the intrinsic muscle force biting FEA scenarios).

Additionally, cranial models assessing the influence of a keratinous rhamphotheca covering the beak of the oviraptorids on the distribution and magnitude of stress in their crania (similarly to Lautenschlager *et al*., 2013) was also tested using material properties from bird beaks ($E = 1.04$ GPa, $\upsilon = 0.40$) (Chen *et al*., 2008; Lautenschlager *et al*., 2013). Hypothetical keratinous coverings were constructed for *Citipati*, *Conchoraptor*, and *Khaan* with a thickness scaled to their cranial lengths (keratin thickness in *Citipati* = 2 mm; *Conchoraptor* = 1.1 mm; *Khaan* = 1.5 mm). The influence of the rhamphotheca on models was primarily tested with bilateral and unilateral muscle driven bite scenarios at the anterior and mid-palate (as the posterior bite position would not be covered by the keratin sheath).

Results

a) Extrinsic scenarios: head-pull, head-shake, and head-twist

Similarly, to the palate loaded comparative strength test, the oviraptorid crania are shown to be comparatively stress resistant in these scenarios modelling head-pull, head-shake, and head-twisting. Oviraptorid oviraptorosaurians perform particularly well in the head-pull scenario and to a slightly lesser extent in the twist-scenario (reversed in *Incisivosaurus*), generally displaying a lower mean stress in one or both scenarios compared with the other theropods (Figs. S1 and S2). Pull is the lower of these two scenarios in the oviraptorids and *Ornithomimus*, twist is the lower in *Incisivosaurus* and *Erlikosaurus*; the two are even in *Allosaurus*. The lowest values for the pull and shake scenarios are found in *Conchoraptor* and *Khaan*. All three oviraptorids, *Citipati* and *Khaan* especially, have very low mean and peak stress with the pull scenario (Fig. S2). Values for pull and shake in *Incisivosaurus* are generally intermediate between the oviraptorids and the other theropods (Fig. S2) and it performs second best in the twisting scenario. The shake scenario produces by far the greatest mean von Mises stress of any of the extrinsic scenarios in every species; it is lowest in *Allosaurus*, but only marginally greater in the four oviraptorosaurians, and notably greater in *Ornithomimus*, followed by *Erlikosaurus* (Fig. S2).

Under the head-pull scenario, oviraptorosaurians experience stress chiefly in the vomer, palatine, pterygoids and jugal (Fig. S1A–D). *Incisivosaurus* differs from the oviraptorid oviraptorosaurians by having more stress at the site of the applied pulling loads (the prominent front teeth, compared with

the edentulous premaxilla of the oviraptorids) and a more stressed premaxilla and nasal (Fig. S1D) which is similar to the non-oviraptorosaurian theropods (Fig. S1M–O).

In the head-shaking scenario, which generally causes higher stresses than the other two extrinsic scenarios, the oviraptorids are typically most stressed at the pterygoid, jugal, quadratojugal, palatine, vomer, and the anteroventral areas of the braincase (Fig. S1E–G). Areas of the premaxilla dorsal to the antorbital fenestra are also highly stressed but less so in *Citipati* (Fig. S1G). The postorbital bar is more stressed in *Khaan* (Fig. S1F) than in *Conchoraptor* (Fig. S1E) or *Citipati* (Fig. S1G). *Incisivosaurus* has stress distributions similar to the oviraptorids but greater magnitudes in the squamosal and lacrimal, and less on the vomer (Fig. S1H). The oviraptorosaurians (Fig. S1E–H) show less stress in their maxilla and through the cranial roof than the other theropods (Fig. S1P–R).

With the head-twisting scenario, the oviraptorids typically show elevated stress at the contribution of the premaxilla to the dorsal edge of the antorbital fenestra and near the contacts of the lacrimal, jugal and maxilla (Fig. S1I–K). *Khaan* and *Citipati* (Fig. S1J,K) also show some stresses at the delicate bony struts of their nasals. *Incisivosaurus* is similar to oviraptorids, but also with slightly elevated stress on the postorbital bar (Fig. S1L), as is the case with *Allosaurus* (Fig. S1S). *Erlikosaurus* and *Ornithomimus* exhibit clearly increased stress around the entire region of the antorbital fenestra and orbit (Fig. S1T,U).

b) Influence of keratinous beak covering

A modelled keratinous rhamphotheca covering part of the premaxilla in *Conchoraptor*, *Khaan*, and *Citipati* was shown to slightly reduce mean von Mises stress in bilateral and unilateral bites at the anterior and middle of the palate (Figs. S3 and S4). This stress reduction only occurs directly beneath the area covered by the rhamphotheca, in the immediate area of the bite points, and in a very minor degree in areas immediately posterior to the covering (no further posterior than the middle of the orbit) chiefly in the premaxilla and lacrimal (Fig. S3). It appears stress in the posterior of the cranium could be slightly increased in the squamosal by the presence of the rhamphotheca in *Khaan* and *Citipati* (Fig. S3). Results of including the rhamphotheca were similar between bilateral and unilateral biting (Fig. S4).

Supplementary References

Chen, P. Y., Lin, A. Y. M., Lin, Y. S., Seki, Y., Stokes, A. G., Peyras, J., Olevsky, E. A., Meyers, M. A. and McKittrick, J. 2008. Structure and mechanical properties of selected biological materials. *Journal of the Mechanical Behavior of Biomedical Materials*, **1**, 208–226.

Dumont, E. R., Grosse, I. R. and Slater, G. J. 2009. Requirements for comparing the performance of finite element models of biological structures. *Journal of Theoretical Biology*, **256**, 96–103.

Lautenschlager, S., Witmer, L. M., Altangerel, P. and Rayfield, E. J. 2013. Edentulism, beaks, and biomechanical innovations in the evolution of theropod dinosaurs. *Proceedings of the National Academy of Sciences*, **110**, 20657–20662.

Montefeltro, F. C., Lautenschlager, S., Godoy, P. L., Ferreira, G. S. and Butler, R. J. 2020. A unique predator in a unique ecosystem: modelling the apex predator within a Late Cretaceous crocodyliform-dominated fauna from Brazil. *Journal of Anatomy*, **237**, 323–333.

Table S1. Surface and volumetric measurements of retrodeformed cranial models used for FEA, information on their mesh geometries, and applied forces (scaled with surface area) for comparative bending test.

**Cranial model (Genus)**	**Surface area (mm²)**	**Volume (mm³)**	**Face count**	**Tetramesh elements**	**Force applied (N)**
Incisivosaurus	23184.9	21500.3	382551	1879557	100.0
Citipati	83100	137631	394540	1830266	358.4
Khaan	39036.9	52091.9	381842	2002012	168.4
Conchoraptor	35336.4	45903.7	380980	2024584	152.4
Allosaurus	804496	6213307	354322	1764510	3469.9
Erlikosaurus	124014	203474	391888	915853	534.9
Ornithomimus	77949	103010	399370	1642940	336.2

Table S2. Comparative von Mises stress and total strain energy results from the analyses modelling bilateral and unilateral bite reaction forces (scaled to relative cranial surface area) on cranial models of oviraptorid oviraptorosaurians *Conchoraptor*, *Khaan*, *Citipati*, along with early diverging oviraptorosaurian *Incisivosaurus,* and *Allosaurus, Erlikosaurus,* and *Ornithomimus* – theropods with more conventional skull morphology. * The top 5% of values are excluded in each dataset to account for artificially high stress values from point loads and nodal constraints. †Total strain energy values reported have undergone an additional correction factor as strain energy scales with volume rather than surface area (Dumont *et al*., 2009).

	**Bilateral loading**			**Unilateral loading**		
	Mean element stress* (MPa)	Peak element stress* (MPa)	Total strain energy† (mJ)	Mean element stress* (MPa)	Peak element stress* (MPa)	Total strain energy† (mJ)
Conchoraptor						
Anterior	2.56	8.05	13.6	2.58	8.08	14.0
Mid-palate	2.19	7.13	10.2	2.37	7.70	11.4
Posterior	1.82	6.39	7.67	1.82	6.40	8.07
Khaan						
Anterior	3.27	10.0	17.5	3.28	10.1	18.0
Mid-palate	2.81	8.81	13.3	2.99	9.43	14.6
Posterior	2.53	8.19	10.8	2.54	8.20	11.3
Citipati						
Anterior	3.67	13.1	22.2	3.73	13.2	22.7
Mid-palate	3.35	11.8	18.0	3.78	12.6	20.4
Posterior	2.81	10.2	12.9	2.81	10.2	13.4
Incisivosaurus						
Anterior	3.88	11.8	21.5	3.87	11.8	21.6
Mid-palate	3.04	9.98	14.6	3.14	10.5	15.7
Posterior	2.78	9.10	11.6	2.92	9.73	13.2
Allosaurus						
Anterior	3.11	11.8	30.5	3.17	11.9	31.0
Mid-palate	2.31	8.83	16.3	2.51	9.41	17.9
Posterior	1.29	5.65	6.26	1.49	6.35	7.85
Erlikosaurus						
Anterior	7.87	23.4	48.3	7.92	24.3	50.6
Mid-palate	5.36	17.3	25.3	5.70	18.9	29.0
Posterior	4.76	15.8	17.4	5.05	17.9	24.3
Ornithomimus						
Anterior	7.86	23.3	49.9	7.91	24.6	52.6
Mid-palate	5.44	17.2	26.6	5.78	19.8	32.8
Posterior	4.67	18.7	29.2	5.15	21.5	41.2

Table S3. Comparative von Mises stress and total strain energy results from the analyses modelling bilateral and unilateral muscle driven bites and three extrinsic scenarios modelling neck muscle driven feeding movement in cranial models of oviraptorid oviraptorosaurians Conchoraptor, Khaan, Citipati, along with early diverging oviraptorosaurian Incisivosaurus, and Allosaurus, Erlikosaurus, and Ornithomimus – theropods with more conventional skull morphology. * The top 5% of values are excluded in each dataset to account for artificially high stress values from point loads and nodal constraints. †Total strain energy values reported have undergone an additional correction factor as strain energy scales with volume rather than surface area (Dumont et al., 2009).

	**Bilateral bite**			**Unilateral bite**				**Extrinsic**		
	Mean element stress* (MPa)	Peak element stress* (MPa)	Total strain energy† (mJ)	Mean element stress* (MPa)	Peak element stress* (MPa)	Total strain energy† (mJ)		Mean element stress* (MPa)	Peak element stress* (MPa)	Total strain energy† (mJ)
Conchoraptor										
Anterior	1.39	4.39	2.10	1.39	4.43	2.20	Pull	0.39	1.92	0.85
Mid-palate	1.36	4.41	2.18	1.42	4.91	2.55	Shake	1.80	6.43	3.82
Posterior	1.41	4.85	2.70	1.40	4.86	2.87	Twist	0.51	2.01	0.34
Khaan										
Anterior	1.53	4.54	3.43	1.53	4.58	3.54	Pull	0.34	1.79	0.43
Mid-palate	1.50	4.50	3.39	1.57	4.88	3.85	Shake	1.77	6.21	4.87
Posterior	1.54	4.90	3.98	1.53	4.91	4.16	Twist	0.43	1.49	0.32
Citipati										
Anterior	1.89	5.92	4.00	1.91	6.02	4.20	Pull	0.35	1.67	0.28
Mid-palate	1.94	6.03	4.31	2.13	6.82	5.66	Shake	1.83	6.68	4.52
Posterior	2.02	6.47	4.93	2.00	6.46	5.28	Twist	0.62	2.04	0.44
Incisivosaurus										
Anterior	1.16	3.26	1.61	1.15	3.27	1.62	Pull	0.69	2.65	0.70
Mid-palate	1.08	3.29	1.63	1.09	3.55	1.83	Shake	2.10	8.52	9.10
Posterior	1.13	3.55	1.87	1.15	3.90	2.21	Twist	0.46	1.75	0.36
Allosaurus										
Anterior	0.78	2.33	1.28	0.78	3.35	1.31	Pull	0.51	2.15	0.79
Mid-palate	0.77	2.34	1.26	0.79	2.56	1.43	Shake	1.63	6.27	9.79
Posterior	0.63	2.26	1.18	0.67	2.63	1.60	Twist	0.50	1.53	0.47
Erlikosaurus										
Anterior	1.43	4.20	1.58	1.42	4.29	1.63	Pull	1.00	3.96	0.89
Mid-palate	1.31	4.08	1.42	1.33	4.32	1.56	Shake	3.51	12.40	10.14
Posterior	1.44	4.60	1.73	1.39	4.89	1.99	Twist	0.86	3.61	0.82
Ornithomimus										
Anterior	0.25	0.78	0.07	0.25	0.80	0.07	Pull	0.95	4.34	1.34
Mid-palate	0.24	0.78	0.06	0.24	0.83	0.07	Shake	3.36	13.00	14.31
Posterior	0.23	0.88	0.08	0.23	0.90	0.09	Twist	1.04	5.18	1.63

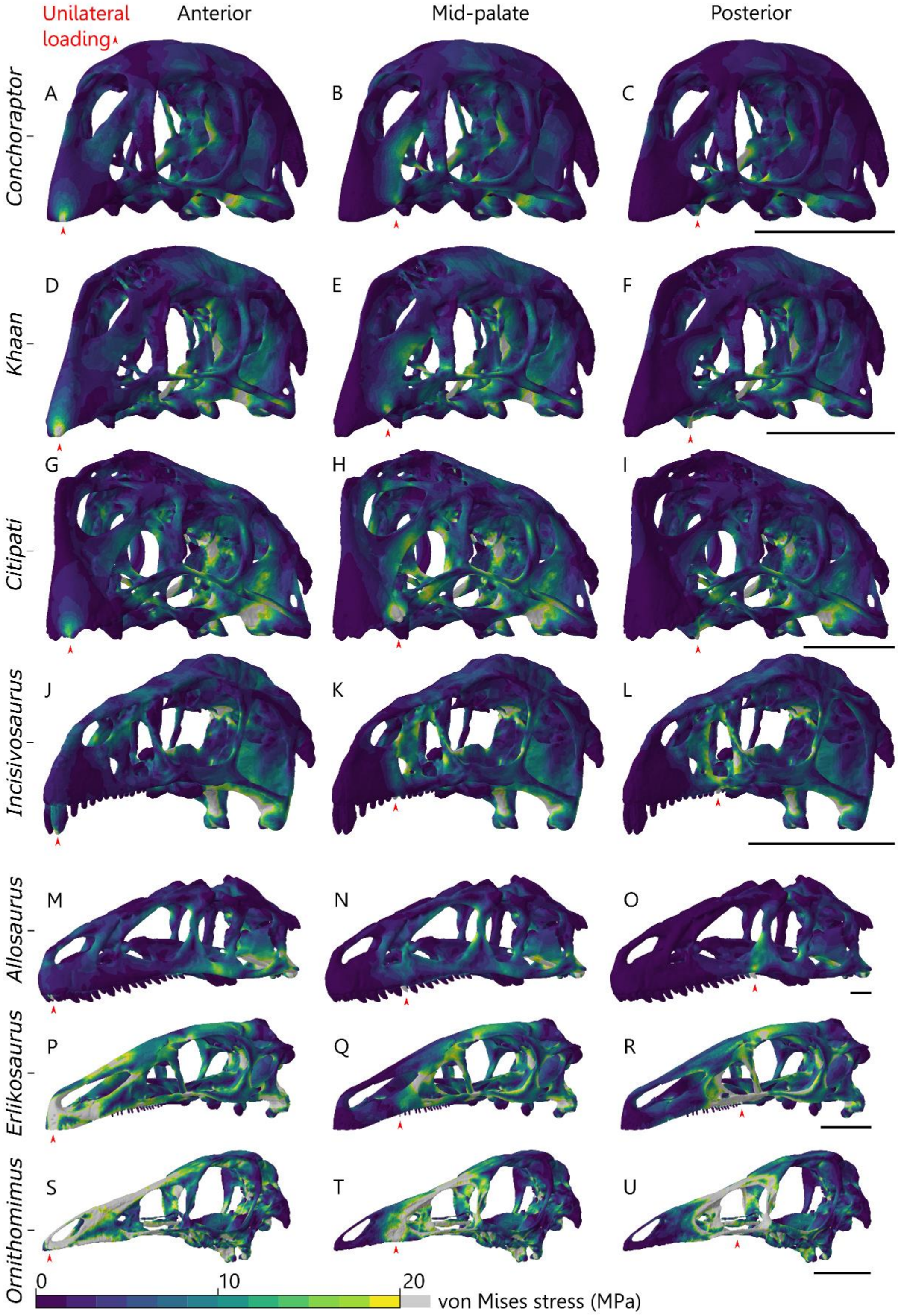

Figure S1. Von Mises stress (MPa) contour plots from FEA of unilaterally applied forces on cranial models of oviraptorosaurians *Conchoraptor* (A–C), *Khaan* (D–F), *Citipati* (G–I), *Incisivosaurus* (J–L), along with other theropod dinosaurs *Allosaurus* (M–O), *Erlikosaurus* (P–R), and *Ornithomimus* (S–U). Applied forces (shown by small red arrow) scaled so ratio of cranial surface area:force applied was identical in all. Forces applied to anterior of the beak/teeth (A, D, G, J, M, P, S), the middle tooth/lateral edge of beak (B, E, H, K, N, Q, T), or the posterior teeth/tooth-like projection on oviraptorid palate (C, F, I, L, O, R, U). All scale bars on the right are 50 mm.

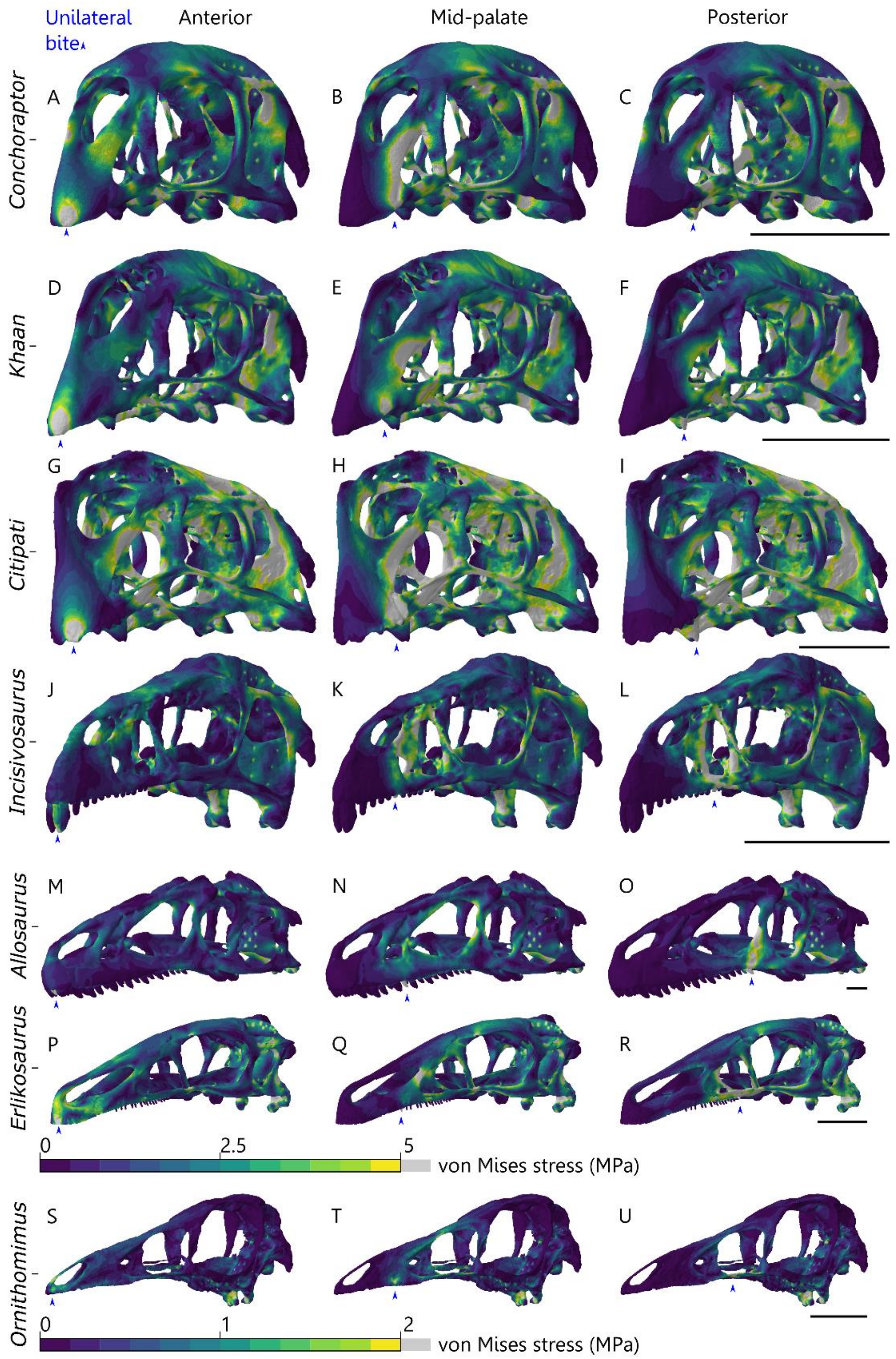

Figure S2. Von Mises stress (MPa) contour plots from FEA using a unilateral bite point constraint and intrinsic muscle forces on cranial models of oviraptorosaurians *Conchoraptor* (A–C), *Khaan* (D–F), *Citipati* (G–I), *Incisivosaurus* (J–L), along with other theropod dinosaurs *Allosaurus* (M–O), *Erlikosaurus* (P–R), and *Ornithomimus* (S–U). Applied forces (shown by small red arrow) scaled so ratio of cranial surface area:force applied was identical in all. Forces applied to anterior of the beak/teeth (A, D, G, J, M, P, S), the middle tooth/lateral edge of beak (B, E, H, K, N, Q, T), or the posterior teeth/tooth-like projection on oviraptorid palate (C, F, I, L, O, R, U). Note *Ornithomimus* is figured at a different stress scale magnitude due to much lower stresses resulting from weaker jaw adductor muscle forces. All scale bars on the right are 50 mm.

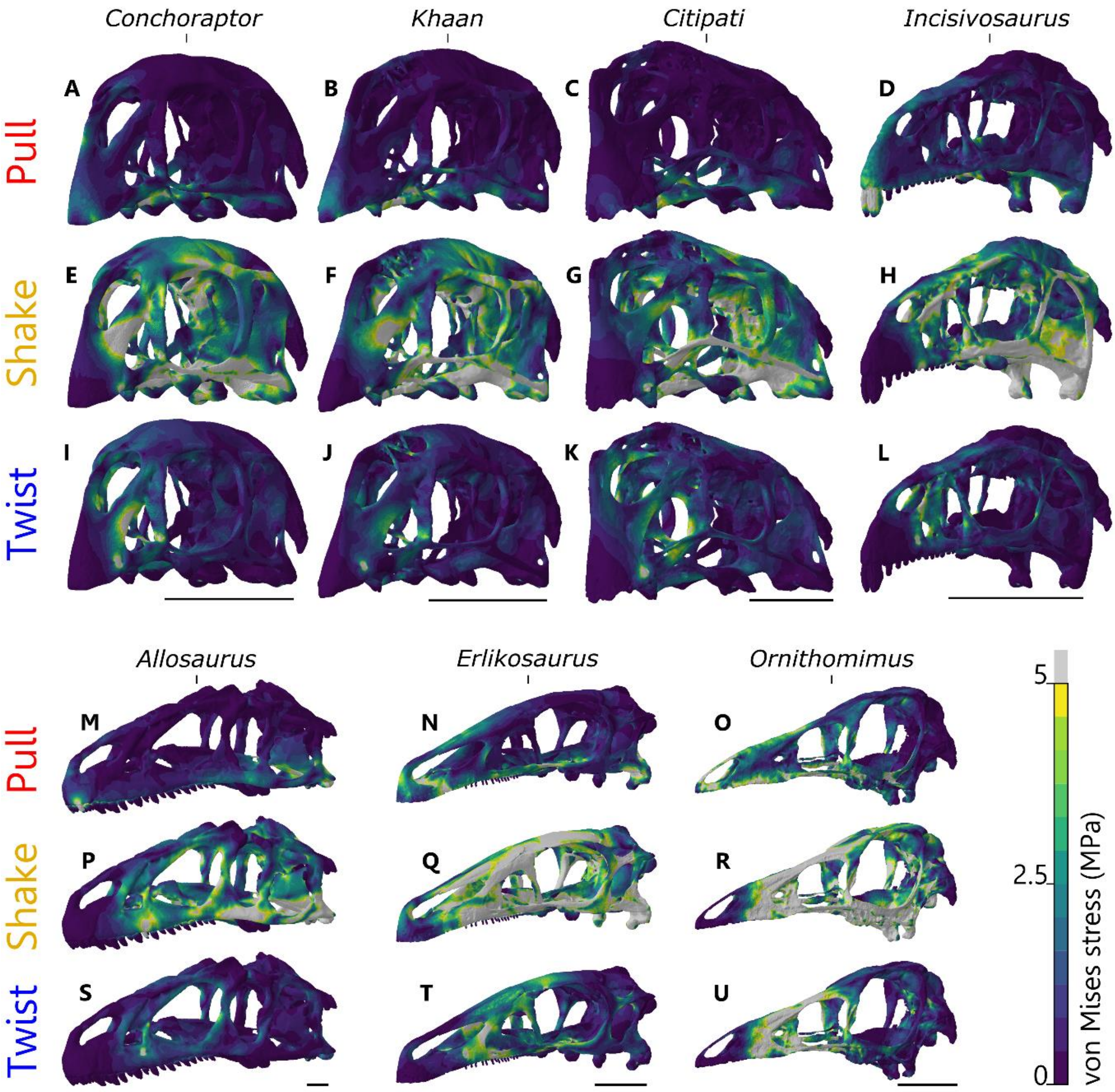

Figure S3. Von Mises stress (MPa) contour plots from FEA modelling head pull-back (A–D, M–O), head shaking (E–H, P–R), and head twisting (I–L, S–U) feeding scenarios using extrinsic applied loads (scaled to cranial surface area) on cranial models of oviraptorosaurians *Conchoraptor* (A,E,I), *Khaan* (B,F,J), *Citipati* (C,G,K), and *Incisivosaurus* (D,H,L), along with other theropod dinosaurs *Allosaurus* (M,P,S), *Erlikosaurus* (N,Q,T), and *Ornithomimus* (O,R,U). All scale bars are 50 mm.

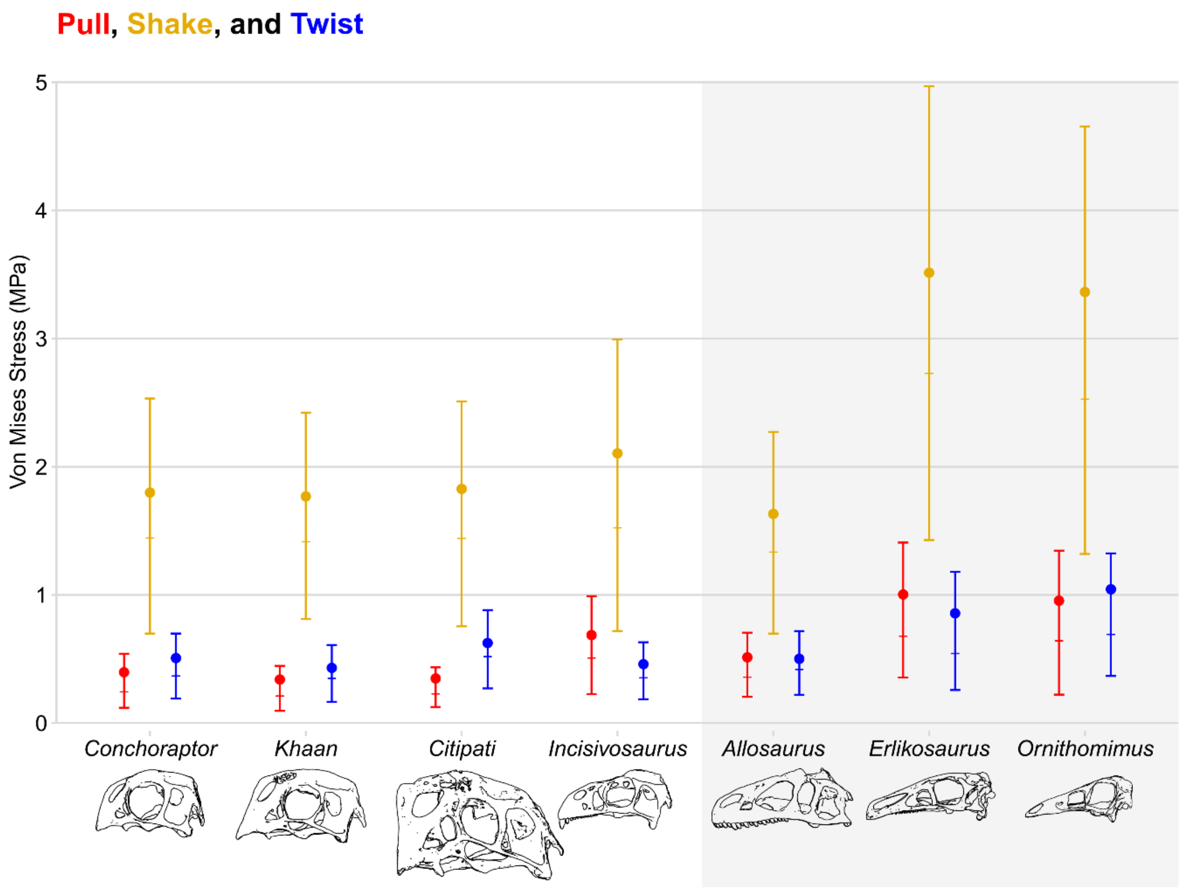

Figure S4. Mean values (points) and whiskers showing first quartile, median, and third quartile of von Mises stress (MPa) from FEA modelling head pull-back (red), head shaking (yellow), and head twisting (blue) feeding scenarios using extrinsic applied loads (scaled to cranial surface area) on cranial models of oviraptorosaurians (white background) *Conchoraptor*, *Khaan*, *Citipati*, and *Incisivosaurus*, along with other theropod dinosaurs (grey background) *Allosaurus*, *Erlikosaurus*, and *Ornithomimus*. The top 5% of values were excluded in each dataset to account for artificially high stress values from point loads and nodal constraints. Outlines of oviraptorosaurian crania are the same relative scale; other theropods are not.

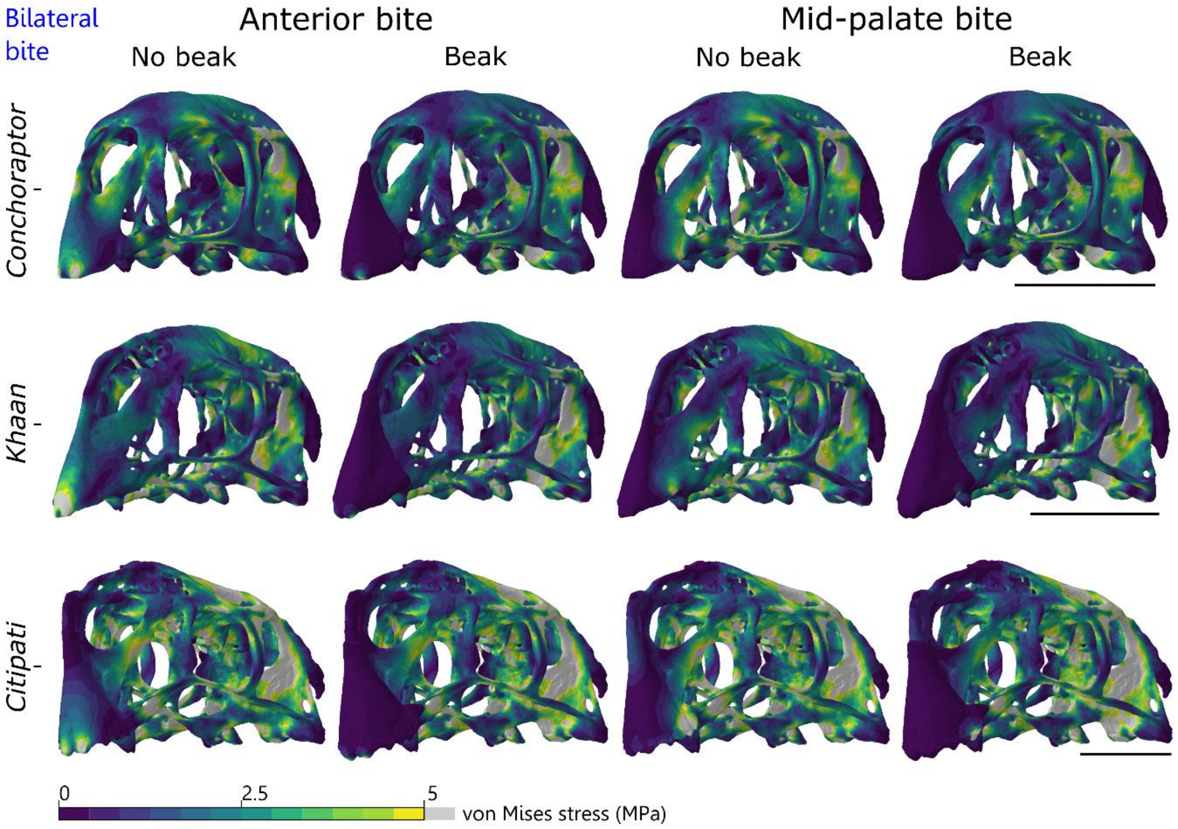

Figure S5. Von Mises stress (MPa) contour plots from FEA using bilateral bite point constraints and intrinsic muscle forces in cranial models of oviraptorosaurians *Conchoraptor* (A–D), *Khaan* (E–H), and *Citipati* (I–L) modelled without (A,C,E,G,I,K) and with (B,D,F,H,J,L) a keratinous rhamphotheca covering the beak. Constraints at bite points shown by small blue arrows at the anterior of the beak (A,B,E,F,I,J) and the lateral edge of beak (C,D,G,H,K,L). All scale bars on the right are 50 mm.

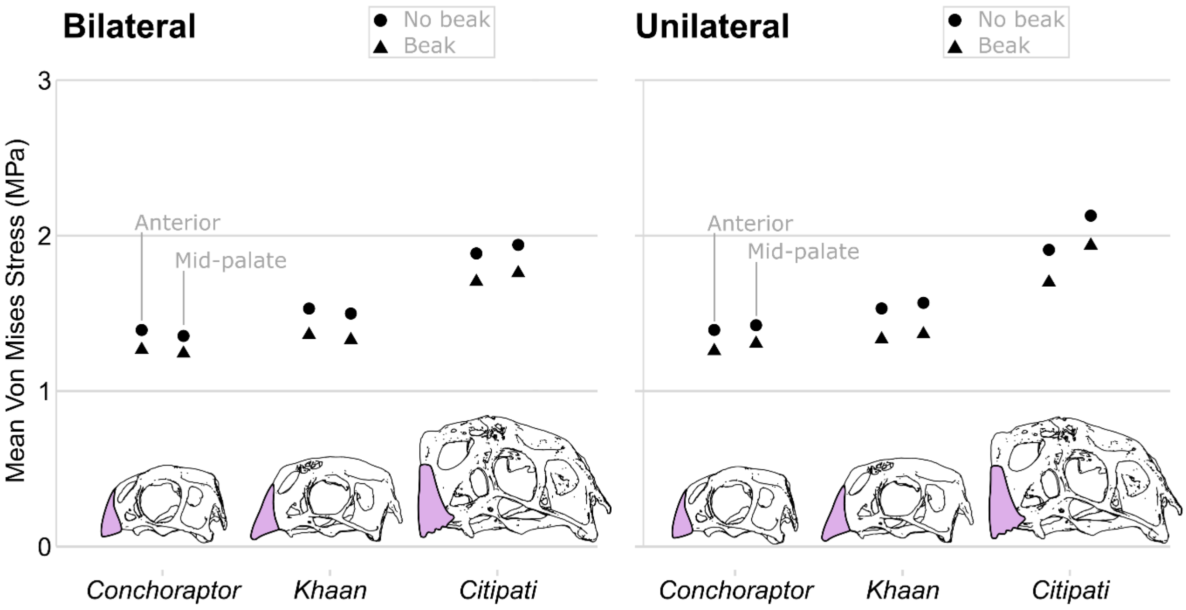

Figure S6. The difference in mean values of von Mises stress (MPa) in cranial models of *Conchoraptor*, *Khaan*, and *Citipati* which either did not (circle point) or did (triangle point) include a keratinous rhamphotheca to the beak in FEA using bilateral (left graph) and unilateral (right graph) bite point constraints and intrinsic muscle forces. Note the small and consistent decrease in mean stress in all scenarios as the keratinous covering only decreased stress in its immediate proximity in the cranium. The top 5% of values were excluded in each dataset to account for artificially high stress values from point loads and nodal constraints. Outlines of crania are the same relative scale and show the extent of the reconstructed rhamphotheca.
