# Supplementary material for: Cranial functional specialisation for strength precedes morphological evolution in Oviraptorosauria
Source: Commun Biol. 2024 Apr 10;7:436. doi: 10.1038/s42003-024-06137-1 (PMC11006937; doi:10.1038/s42003-024-06137-1)
Supplement: Supplementary file 3 — Reporting Summary [file 42003_2024_6137_MOESM3_ESM.pdf]

Corresponding author(s): Luke E. Meade

Last updated by author(s): Jan 26, 2024

Reporting Summary

Nature Portfolio wishes to improve the reproducibility of the work that we publish. This form provides structure for consistency and transparency in reporting. For further information on Nature Portfolio policies, see our Editorial Policies and the Editorial Policy Checklist.

Statistics

For all statistical analyses, confirm that the following items are present in the figure legend, table legend, main text, or Methods section.

n/a	Confirmed	
☐	☒	The exact sample size (*n*) for each experimental group/condition, given as a discrete number and unit of measurement
☐	☒	A statement on whether measurements were taken from distinct samples or whether the same sample was measured repeatedly
☒	☐	The statistical test(s) used AND whether they are one- or two-sided<br>*Only common tests should be described solely by name; describe more complex techniques in the Methods section.*
☒	☐	A description of all covariates tested
☒	☐	A description of any assumptions or corrections, such as tests of normality and adjustment for multiple comparisons
☒	☐	A full description of the statistical parameters including central tendency (e.g. means) or other basic estimates (e.g. regression coefficient) AND variation (e.g. standard deviation) or associated estimates of uncertainty (e.g. confidence intervals)
☒	☐	For null hypothesis testing, the test statistic (e.g. *F*, *t*, *r*) with confidence intervals, effect sizes, degrees of freedom and *P* value noted<br>*Give P values as exact values whenever suitable.*
☒	☐	For Bayesian analysis, information on the choice of priors and Markov chain Monte Carlo settings
☒	☐	For hierarchical and complex designs, identification of the appropriate level for tests and full reporting of outcomes
☒	☐	Estimates of effect sizes (e.g. Cohen's *d*, Pearson's *r*), indicating how they were calculated

Our web collection on statistics for biologists contains articles on many of the points above.

Software and code

Policy information about availability of computer code

Data collection	Avizo Lite (version 9.3.0); Blender (version 2.9.0)
Data analysis	Hypermesh (version 13.0.110); Abaqus (version 6.14)

For manuscripts utilizing custom algorithms or software that are central to the research but not yet described in published literature, software must be made available to editors and reviewers. We strongly encourage code deposition in a community repository (e.g. GitHub). See the Nature Portfolio guidelines for submitting code & software for further information.

Data

Policy information about availability of data

All manuscripts must include a data availability statement. This statement should provide the following information, where applicable:

- Accession codes, unique identifiers, or web links for publicly available datasets
- A description of any restrictions on data availability
- For clinical datasets or third party data, please ensure that the statement adheres to our policy

The datasets, including cranial 3D models and files for finite element analysis, generated and analysed during the current study are available from the Zenodo data repository for download at: https://doi.org/10.5281/zenodo.10379298.

Research involving human participants, their data, or biological material

Policy information about studies with human participants or human data. See also policy information about sex, gender (identity/presentation), and sexual orientation and race, ethnicity and racism.

Reporting on sex and gender	n/a
Reporting on race, ethnicity, or other socially relevant groupings	n/a
Population characteristics	n/a
Recruitment	n/a
Ethics oversight	n/a

Note that full information on the approval of the study protocol must also be provided in the manuscript.

Field-specific reporting

Please select the one below that is the best fit for your research. If you are not sure, read the appropriate sections before making your selection.

☐ Life sciences ☐ Behavioural & social sciences ☒ Ecological, evolutionary & environmental sciences

For a reference copy of the document with all sections, see nature.com/documents/nr-reporting-summary-flat.pdf

Ecological, evolutionary & environmental sciences study design

All studies must disclose on these points even when the disclosure is negative.

Study description	Finite element analysis study of digital dinosaur 3D cranial models
Research sample	7 crania from 7 different genera representing oviraptorosaurians compared with other herbivorous theropods and Allosaurus
Sampling strategy	Study based on CT-datasets generated for previous studies, relevant datasets were sourced from collaborators
Data collection	Models segmented from CT-data and FEA scenarios set up with muscle force vectors based on published literature
Timing and spatial scale	n/a
Data exclusions	n/a
Reproducibility	Files for all analyses and raw data provided in data availability statement
Randomization	n/a - One model representing each genera - most genera only known from single specimen
Blinding	n/a

Did the study involve field work? ☐ Yes ☒ No

Reporting for specific materials, systems and methods

We require information from authors about some types of materials, experimental systems and methods used in many studies. Here, indicate whether each material, system or method listed is relevant to your study. If you are not sure if a list item applies to your research, read the appropriate section before selecting a response.

Materials & experimental systems

n/a	Involved in the study
☒	☐ Antibodies
☒	☐ Eukaryotic cell lines
☐	☒ Palaeontology and archaeology
☒	☐ Animals and other organisms
☒	☐ Clinical data
☒	☐ Dual use research of concern
☒	☐ Plants

Methods

n/a	Involved in the study
☒	☐ ChIP-seq
☒	☐ Flow cytometry
☒	☐ MRI-based neuroimaging

Palaeontology and Archaeology

Specimen provenance: Specimens were studied digitally based on previously published CT-datasets

Specimen deposition: Incisivosaurus (IVPP V13326) - Institute of Vertebrate Paleontology and Paleoanthropology (IVPP), Chinese Academy of Sciences.
Citipati (MPC-D 100/798), Khaan (MPC-D 100/973), and Conchoraptor (MPC-D 100/3006 - Institute of Paleontology in Ulaanbaatar, Mongolia.
Conchoraptor (ZPAL Mg-D I/95) - Institute of Paleobiology Polish Academy of Sciences Warsaw Poland.

Dating methods: n/a

☐ Tick this box to confirm that the raw and calibrated dates are available in the paper or in Supplementary Information.

Ethics oversight: n/a - studied digitally based on previously published CT-datasets

Note that full information on the approval of the study protocol must also be provided in the manuscript.

Plants

Seed stocks: n/a

Novel plant genotypes: n/a

Authentication: n/a
